# Surgical Treatment of Cutaneous Anthrax

**DOI:** 10.1590/0037-8682-0062-2019

**Published:** 2019-12-20

**Authors:** Changsong Zhao, Qiang Zhang, Yao Zhang

**Affiliations:** 1 Capital Medical University, Beijing Ditan Hospital, Department of Orthopedics, Beijing, China.

**FIGURE 1:**
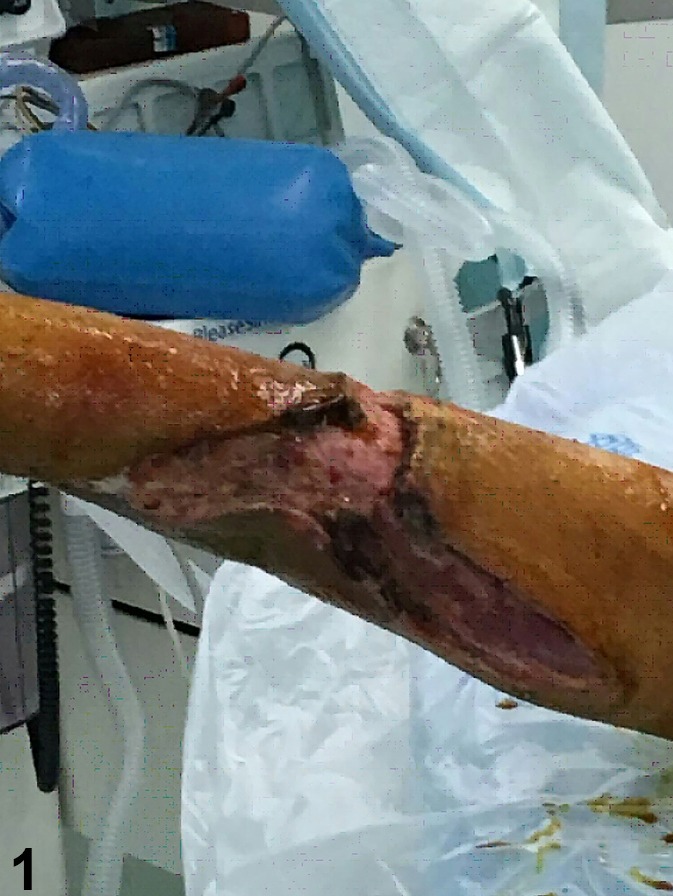
A patient with cutaneous anthrax was diagnosed with compartment syndrome at another hospital. He had lesion incision, tension reduction, and vacuum drainage. The incised wound can be seen.

**FIGURE 2:**
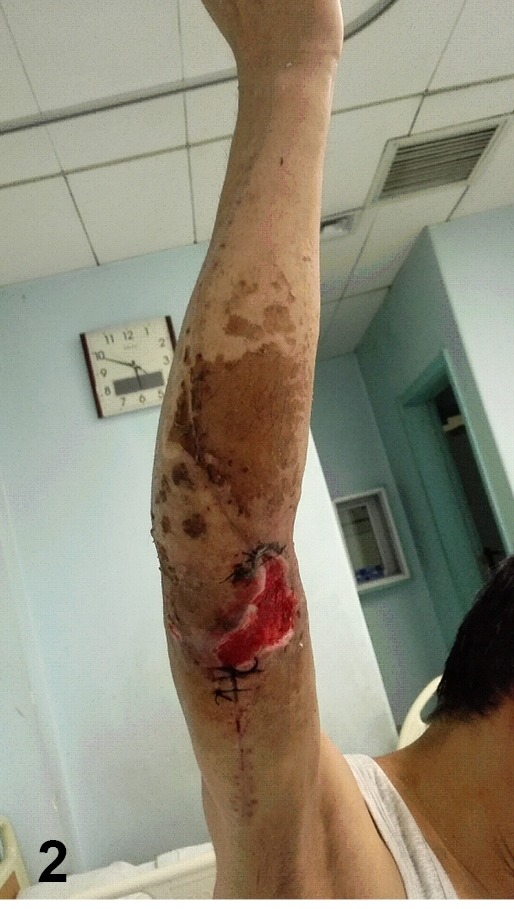
Triangle-shaped skin necrosis developed after suture removal, and a large skin defect formed after debridement.

**FIGURE 3:**
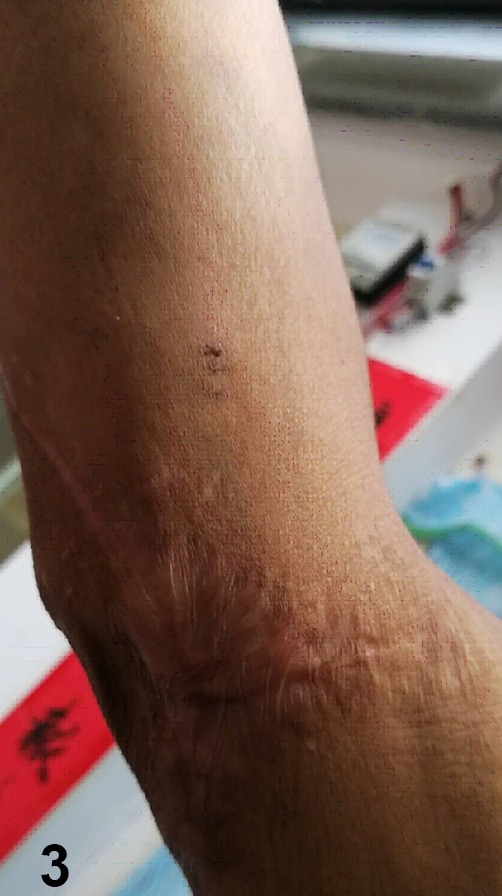
The wound healed well after one month of dressing treatment.

A 45-year-old male patient came into contact with a dead cow. Subsequently, a cutaneous rash appeared in his right upper extremity, which gradually increased in size and ulcerated^1^. His temperature was elevated up to 40°C. His arm became severely red, swollen, and painful with high tension and high skin temperature. Laboratory tests revealed white blood cell count of 19.88×10^9^/L, 92.5% neutrophils, procalcitonin level of 8.79 ng/mL, and interleukin-6 level of 277.7 pg/mL. The patient had lesion incision and tension reduction, followed by vacuum drainage (Figure 1) and antibiotic therapy with meropenem at another hospital. At our hospital, he received clindamycin and levofloxacin treatments and four weeks of nutritional support. Eventually, the C-reactive protein level, white blood cell count, neutrophil percentage, and temperature returned to normal. The *Bacillus anthracis* nucleic acid was positive in the wound. After four days of hospital stay, debridement and suture surgery were performed. Triangle-shaped skin necrosis developed after suture removal. A large skin defect formed after debridement.

 (Figure 2). The patient refused to receive a transplanted flap. The wound secretion test was negative for *B. anthracis* nucleic acid. After one month of dressing treatment, the wound healed. 

(Figure 3). The main treatment for cutaneous anthrax is antibiotics. Compartment syndrome should be treated with fasciotomy^2^ ^-^ ^3^.
